# 3-Iodo-8β,9α,14α-estra-1,3,5(10)-trien-17-one

**DOI:** 10.1107/S1600536809014603

**Published:** 2009-05-07

**Authors:** Kamal Aziz Ketuly, A. Hamid A. Hadi, Seik Weng Ng

**Affiliations:** a Department of Chemistry, University of Malaya, 50603 Kuala Lumpur, Malaysia

## Abstract

In the title compound, C_18_H_21_IO, the cyclo­hexane ring adopts a chair conformation, whereas the cyclo­pentane ring and the ten-membered tetra­line portions each adopt an envelope conformation. For the five-membered ring, the methine C atom deviates by 0.638 (4) Å (r.m.s. of the four other atoms is 0.005 Å) and for the ten-membered ring, the methine C atom constituting the flap deviates by 0.671 (3) Å (r.m.s. of the other nine atoms is 0.066 Å).

## Related literature

There are only a few crystal structure reports of similar compounds; for the methoxyl-substituted derivative, see: Herrmann *et al.* (2006 ▶). For the synthesis of the 3-amino-substituted reagent, see: Conrow & Bernstein (1968 ▶).
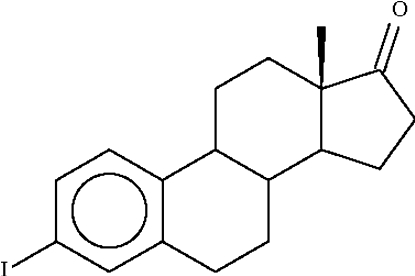


## Experimental

### 

#### Crystal data

C_18_H_21_IO
*M* *_r_* = 380.25
Orthorhombic, 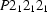

*a* = 9.9636 (2) Å
*b* = 10.5246 (2) Å
*c* = 14.3535 (2) Å
*V* = 1505.15 (5) Å^3^
*Z* = 4
Mo *K*α radiation
μ = 2.12 mm^−1^
*T* = 100 K
0.20 × 0.15 × 0.10 mm

#### Data collection

Bruker SMART APEX diffractometer
Absorption correction: multi-scan (*SADABS*; Sheldrick, 1996 ▶) *T*_min_ = 0.636, *T*_max_ = 0.746 (expected range = 0.690–0.809)
10547 measured reflections
3452 independent reflections
3353 reflections with *I* > 2σ(*I*)
*R*_int_ = 0.021

#### Refinement

*R*[*F* ^2^ > 2σ(*F* ^2^)] = 0.018
*wR*(*F* ^2^) = 0.045
*S* = 1.03
3452 reflections
181 parameters
H-atom parameters constrained
Δρ_max_ = 0.47 e Å^−3^
Δρ_min_ = −0.39 e Å^−3^
Absolute structure: Flack (1983 ▶), 1473 Friedel pairs
Flack parameter: 0.01 (2)

### 

Data collection: *APEX2* (Bruker, 2008 ▶); cell refinement: *SAINT* (Bruker, 2008 ▶); data reduction: *SAINT*; program(s) used to solve structure: *SHELXS97* (Sheldrick, 2008 ▶); program(s) used to refine structure: *SHELXL97* (Sheldrick, 2008 ▶); molecular graphics: *X-SEED* (Barbour, 2001 ▶); software used to prepare material for publication: *publCIF* (Westrip, 2009 ▶).

## Supplementary Material

Crystal structure: contains datablocks global, I. DOI: 10.1107/S1600536809014603/tk2435sup1.cif

Structure factors: contains datablocks I. DOI: 10.1107/S1600536809014603/tk2435Isup2.hkl

Additional supplementary materials:  crystallographic information; 3D view; checkCIF report

## Figures and Tables

**Fig. 1.**
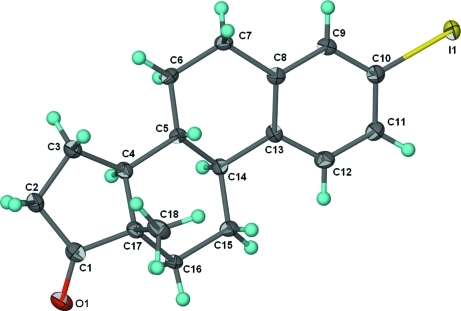
Displacement ellipsoid plot (Barbour, 2001) of C18H21IO at the 70% probability level. H atoms are drawn as spheres of arbitrary radius.

## supplementary crystallographic information

### Experimental

The reactant, 3-amino-estra-1,3,5(1)-trien-17-one, was synthesized by using a literature procedure (Conrow & Bernstein, 1968). The compound (390 mg) was dissolved in hydrobromic acid (54% w/v). Concentrated sulfuric acid (2 ml) and water (4 ml) were added. The solution was cooled in an ice-bath. Sodium nitrite (147 mg) in water (2 ml) was added followed by the addition of excess potassium iodide (1.02 g) dissolved in water (4 ml).

The solution was filtered and the gummy product collected and dissolved in ether–ethyl acetate. The solvent was removed and the crude product (537 mg) chromatographed on a silica-gel (40 g) column. The compound was eluted by chloroform–ethyl acetate (3:1 v/v). The second fraction (465 mg) was a tan glassy material. This was recrystallized from chloroform, methanol and ethyl acetate to give single crystals.

### Refinement

Hydrogen atoms were placed at calculated positions (C–H 0.95–1.00 Å) and were treated as riding on their parent carbon atoms, with *U*(H) set to 1.2–1.5 times *U*_eq_(*C*).

### Crystal data

**Table:**

C_18_H_21_IO	*F*(000) = 760
*M**_r_* = 380.25	*D*_x_ = 1.678 Mg m^−^^3^
Orthorhombic, *P*2_1_2_1_2_1_	Mo *K*α radiation, λ = 0.71073 Å
Hall symbol: P 2ac 2ab	Cell parameters from 6954 reflections
*a* = 9.9636 (2) Å	θ = 2.4–28.3°
*b* = 10.5246 (2) Å	µ = 2.12 mm^−^^1^
*c* = 14.3535 (2) Å	*T* = 100 K
*V* = 1505.15 (5) Å^3^	Irregular block, colorless
*Z* = 4	0.20 × 0.15 × 0.10 mm

### Data collection

**Table:**

Bruker SMART APEX diffractometer	3452 independent reflections
Radiation source: fine-focus sealed tube	3353 reflections with *I* > 2σ(*I*)
graphite	*R*_int_ = 0.021
ω scans	θ_max_ = 27.5°, θ_min_ = 2.4°
Absorption correction: multi-scan (*SADABS*; Sheldrick, 1996)	*h* = −12→12
*T*_min_ = 0.636, *T*_max_ = 0.746	*k* = −13→13
10547 measured reflections	*l* = −18→18

### Refinement

**Table:**

Refinement on *F*^2^	Secondary atom site location: difference Fourier map
Least-squares matrix: full	Hydrogen site location: inferred from neighbouring sites
*R*[*F*^2^ > 2σ(*F*^2^)] = 0.018	H-atom parameters constrained
*wR*(*F*^2^) = 0.045	*w* = 1/[σ^2^(*F*_o_^2^) + (0.0262*P*)^2^] where *P* = (*F*_o_^2^ + 2*F*_c_^2^)/3
*S* = 1.03	(Δ/σ)_max_ = 0.001
3452 reflections	Δρ_max_ = 0.47 e Å^−^^3^
181 parameters	Δρ_min_ = −0.39 e Å^−^^3^
0 restraints	Absolute structure: Flack (1983), 1473 Friedel pairs
Primary atom site location: structure-invariant direct methods	Flack parameter: 0.01 (2)

### Fractional atomic coordinates and isotropic or equivalent isotropic displacement parameters (Å^2^)

**Table:**

	*x*	*y*	*z*	*U*_iso_*/*U*_eq_	
I1	0.398911 (14)	1.187407 (14)	0.171916 (11)	0.01746 (5)	
O1	0.05863 (18)	0.21965 (16)	0.45415 (13)	0.0230 (4)	
C1	0.0408 (2)	0.3314 (2)	0.46921 (16)	0.0164 (5)	
C2	−0.0646 (3)	0.3860 (2)	0.53518 (19)	0.0192 (6)	
H2A	−0.0492	0.3557	0.5996	0.023*	
H2B	−0.1558	0.3604	0.5155	0.023*	
C3	−0.0483 (3)	0.5320 (2)	0.52963 (18)	0.0185 (5)	
H3A	−0.1362	0.5755	0.5333	0.022*	
H3B	0.0108	0.5641	0.5798	0.022*	
C4	0.0166 (2)	0.5494 (2)	0.43324 (16)	0.0133 (5)	
H4	−0.0548	0.5300	0.3864	0.016*	
C5	0.0771 (2)	0.6760 (2)	0.40501 (15)	0.0117 (4)	
H5	0.1566	0.6930	0.4456	0.014*	
C6	−0.0183 (2)	0.7883 (2)	0.41237 (16)	0.0146 (5)	
H6A	−0.0606	0.7891	0.4748	0.018*	
H6B	−0.0903	0.7799	0.3652	0.018*	
C7	0.0573 (2)	0.9119 (2)	0.39693 (17)	0.0143 (5)	
H7A	0.1071	0.9338	0.4544	0.017*	
H7B	−0.0081	0.9808	0.3848	0.017*	
C8	0.1562 (2)	0.9044 (2)	0.31532 (16)	0.0126 (5)	
C9	0.2161 (2)	1.0179 (2)	0.28663 (17)	0.0139 (5)	
H9	0.1950	1.0952	0.3175	0.017*	
C10	0.3063 (2)	1.0176 (2)	0.21308 (17)	0.0144 (5)	
C11	0.3359 (2)	0.9057 (2)	0.16548 (19)	0.0169 (5)	
H11	0.3958	0.9061	0.1140	0.020*	
C12	0.2762 (2)	0.7942 (2)	0.19494 (15)	0.0168 (5)	
H12	0.2962	0.7176	0.1628	0.020*	
C13	0.1868 (2)	0.7901 (2)	0.27081 (16)	0.0129 (5)	
C14	0.1260 (2)	0.6646 (2)	0.30264 (15)	0.0128 (5)	
H14	0.0443	0.6503	0.2636	0.015*	
C15	0.2184 (3)	0.5497 (2)	0.28692 (18)	0.0176 (5)	
H15A	0.2341	0.5399	0.2192	0.021*	
H15B	0.3061	0.5670	0.3167	0.021*	
C16	0.1628 (2)	0.4241 (2)	0.32552 (19)	0.0162 (5)	
H16A	0.0849	0.3967	0.2876	0.019*	
H16B	0.2326	0.3574	0.3215	0.019*	
C17	0.1194 (2)	0.4408 (2)	0.42701 (15)	0.0133 (5)	
C18	0.2423 (2)	0.4595 (2)	0.49110 (18)	0.0191 (5)	
H18A	0.2991	0.5274	0.4661	0.029*	
H18B	0.2939	0.3804	0.4941	0.029*	
H18C	0.2119	0.4827	0.5538	0.029*	

### Atomic displacement parameters (Å^2^)

**Table:**

	*U*^11^	*U*^22^	*U*^33^	*U*^12^	*U*^13^	*U*^23^
I1	0.01720 (7)	0.01525 (7)	0.01993 (8)	−0.00127 (6)	0.00318 (6)	0.00288 (7)
O1	0.0296 (10)	0.0126 (9)	0.0269 (10)	−0.0016 (7)	0.0042 (8)	−0.0013 (7)
C1	0.0185 (11)	0.0152 (12)	0.0155 (11)	−0.0005 (10)	−0.0024 (9)	0.0012 (10)
C2	0.0233 (13)	0.0143 (12)	0.0199 (13)	−0.0021 (10)	0.0064 (10)	0.0013 (10)
C3	0.0237 (12)	0.0134 (12)	0.0185 (12)	−0.0014 (9)	0.0064 (10)	−0.0010 (10)
C4	0.0161 (11)	0.0123 (11)	0.0115 (11)	−0.0015 (9)	0.0001 (9)	−0.0008 (9)
C5	0.0114 (10)	0.0119 (10)	0.0118 (10)	0.0016 (9)	−0.0003 (8)	−0.0013 (9)
C6	0.0148 (11)	0.0152 (12)	0.0138 (11)	0.0014 (9)	0.0031 (9)	−0.0015 (9)
C7	0.0186 (12)	0.0119 (11)	0.0124 (11)	0.0014 (9)	0.0029 (9)	−0.0016 (9)
C8	0.0114 (9)	0.0138 (11)	0.0125 (12)	0.0017 (8)	−0.0034 (9)	0.0005 (10)
C9	0.0148 (12)	0.0131 (11)	0.0137 (11)	0.0008 (9)	−0.0031 (9)	−0.0006 (9)
C10	0.0124 (10)	0.0152 (11)	0.0158 (11)	0.0009 (9)	−0.0013 (9)	0.0014 (9)
C11	0.0182 (11)	0.0187 (11)	0.0137 (11)	0.0004 (9)	0.0030 (10)	−0.0012 (11)
C12	0.0221 (12)	0.0153 (12)	0.0131 (11)	0.0011 (9)	0.0023 (9)	−0.0023 (9)
C13	0.0126 (10)	0.0126 (12)	0.0135 (11)	−0.0009 (9)	−0.0020 (8)	0.0016 (9)
C14	0.0141 (11)	0.0125 (11)	0.0116 (11)	−0.0012 (8)	0.0010 (8)	−0.0008 (8)
C15	0.0232 (13)	0.0132 (12)	0.0165 (12)	0.0008 (10)	0.0080 (10)	−0.0022 (10)
C16	0.0210 (11)	0.0095 (10)	0.0181 (11)	−0.0012 (9)	0.0067 (11)	−0.0038 (12)
C17	0.0161 (12)	0.0115 (10)	0.0124 (11)	−0.0023 (10)	0.0002 (9)	−0.0016 (9)
C18	0.0185 (11)	0.0137 (11)	0.0250 (13)	0.0014 (9)	−0.0075 (10)	0.0028 (11)

### Geometric parameters (Å, °)

**Table:**

I1—C10	2.097 (2)	C8—C9	1.397 (3)
O1—C1	1.209 (3)	C8—C13	1.396 (3)
C1—C2	1.526 (3)	C9—C10	1.386 (3)
C1—C17	1.518 (3)	C9—H9	0.9500
C2—C3	1.547 (3)	C10—C11	1.393 (3)
C2—H2A	0.9900	C11—C12	1.382 (3)
C2—H2B	0.9900	C11—H11	0.9500
C3—C4	1.538 (3)	C12—C13	1.407 (3)
C3—H3A	0.9900	C12—H12	0.9500
C3—H3B	0.9900	C13—C14	1.524 (3)
C4—C5	1.517 (3)	C14—C15	1.537 (3)
C4—C17	1.539 (3)	C14—H14	1.0000
C4—H4	1.0000	C15—C16	1.537 (3)
C5—C6	1.520 (3)	C15—H15A	0.9900
C5—C14	1.553 (3)	C15—H15B	0.9900
C5—H5	1.0000	C16—C17	1.529 (3)
C6—C7	1.520 (3)	C16—H16A	0.9900
C6—H6A	0.9900	C16—H16B	0.9900
C6—H6B	0.9900	C17—C18	1.544 (3)
C7—C8	1.533 (3)	C18—H18A	0.9800
C7—H7A	0.9900	C18—H18B	0.9800
C7—H7B	0.9900	C18—H18C	0.9800
			
O1—C1—C2	125.3 (2)	C8—C9—H9	120.0
O1—C1—C17	126.2 (2)	C9—C10—C11	120.9 (2)
C2—C1—C17	108.5 (2)	C9—C10—I1	119.85 (18)
C1—C2—C3	105.6 (2)	C11—C10—I1	119.26 (18)
C1—C2—H2A	110.6	C12—C11—C10	118.4 (2)
C3—C2—H2A	110.6	C12—C11—H11	120.8
C1—C2—H2B	110.6	C10—C11—H11	120.8
C3—C2—H2B	110.6	C11—C12—C13	122.3 (2)
H2A—C2—H2B	108.7	C11—C12—H12	118.8
C4—C3—C2	102.1 (2)	C13—C12—H12	118.8
C4—C3—H3A	111.4	C8—C13—C12	117.8 (2)
C2—C3—H3A	111.4	C8—C13—C14	121.5 (2)
C4—C3—H3B	111.4	C12—C13—C14	120.7 (2)
C2—C3—H3B	111.4	C13—C14—C15	113.55 (18)
H3A—C3—H3B	109.2	C13—C14—C5	109.98 (19)
C5—C4—C3	120.79 (19)	C15—C14—C5	112.83 (19)
C5—C4—C17	111.87 (18)	C13—C14—H14	106.7
C3—C4—C17	104.08 (19)	C15—C14—H14	106.7
C5—C4—H4	106.4	C5—C14—H14	106.7
C3—C4—H4	106.4	C16—C15—C14	114.06 (19)
C17—C4—H4	106.4	C16—C15—H15A	108.7
C4—C5—C6	114.57 (18)	C14—C15—H15A	108.7
C4—C5—C14	108.02 (18)	C16—C15—H15B	108.7
C6—C5—C14	108.79 (18)	C14—C15—H15B	108.7
C4—C5—H5	108.4	H15A—C15—H15B	107.6
C6—C5—H5	108.4	C17—C16—C15	110.27 (19)
C14—C5—H5	108.4	C17—C16—H16A	109.6
C5—C6—C7	110.23 (18)	C15—C16—H16A	109.6
C5—C6—H6A	109.6	C17—C16—H16B	109.6
C7—C6—H6A	109.6	C15—C16—H16B	109.6
C5—C6—H6B	109.6	H16A—C16—H16B	108.1
C7—C6—H6B	109.6	C1—C17—C16	116.02 (19)
H6A—C6—H6B	108.1	C1—C17—C4	101.32 (18)
C6—C7—C8	112.71 (19)	C16—C17—C4	109.19 (19)
C6—C7—H7A	109.1	C1—C17—C18	105.57 (19)
C8—C7—H7A	109.1	C16—C17—C18	111.01 (19)
C6—C7—H7B	109.1	C4—C17—C18	113.48 (19)
C8—C7—H7B	109.1	C17—C18—H18A	109.5
H7A—C7—H7B	107.8	C17—C18—H18B	109.5
C9—C8—C13	120.6 (2)	H18A—C18—H18B	109.5
C9—C8—C7	117.1 (2)	C17—C18—H18C	109.5
C13—C8—C7	122.3 (2)	H18A—C18—H18C	109.5
C10—C9—C8	119.9 (2)	H18B—C18—H18C	109.5
C10—C9—H9	120.0		
			
O1—C1—C2—C3	−179.5 (2)	C8—C13—C14—C15	−148.7 (2)
C17—C1—C2—C3	1.1 (3)	C12—C13—C14—C15	31.4 (3)
C1—C2—C3—C4	24.3 (3)	C8—C13—C14—C5	−21.2 (3)
C2—C3—C4—C5	−167.6 (2)	C12—C13—C14—C5	158.9 (2)
C2—C3—C4—C17	−41.0 (2)	C4—C5—C14—C13	179.61 (18)
C3—C4—C5—C6	−55.3 (3)	C6—C5—C14—C13	54.7 (2)
C17—C4—C5—C6	−178.27 (19)	C4—C5—C14—C15	−52.5 (2)
C3—C4—C5—C14	−176.7 (2)	C6—C5—C14—C15	−177.42 (19)
C17—C4—C5—C14	60.3 (2)	C13—C14—C15—C16	175.6 (2)
C4—C5—C6—C7	171.42 (19)	C5—C14—C15—C16	49.6 (3)
C14—C5—C6—C7	−67.6 (2)	C14—C15—C16—C17	−50.9 (3)
C5—C6—C7—C8	43.4 (3)	O1—C1—C17—C16	36.6 (3)
C6—C7—C8—C9	170.8 (2)	C2—C1—C17—C16	−144.0 (2)
C6—C7—C8—C13	−9.4 (3)	O1—C1—C17—C4	154.7 (2)
C13—C8—C9—C10	0.3 (3)	C2—C1—C17—C4	−25.9 (2)
C7—C8—C9—C10	−179.9 (2)	O1—C1—C17—C18	−86.8 (3)
C8—C9—C10—C11	1.6 (4)	C2—C1—C17—C18	92.6 (2)
C8—C9—C10—I1	−178.76 (17)	C15—C16—C17—C1	169.9 (2)
C9—C10—C11—C12	−1.8 (4)	C15—C16—C17—C4	56.3 (2)
I1—C10—C11—C12	178.55 (18)	C15—C16—C17—C18	−69.6 (2)
C10—C11—C12—C13	0.2 (4)	C5—C4—C17—C1	173.37 (18)
C9—C8—C13—C12	−1.9 (3)	C3—C4—C17—C1	41.4 (2)
C7—C8—C13—C12	178.3 (2)	C5—C4—C17—C16	−63.7 (2)
C9—C8—C13—C14	178.2 (2)	C3—C4—C17—C16	164.26 (19)
C7—C8—C13—C14	−1.6 (3)	C5—C4—C17—C18	60.7 (3)
C11—C12—C13—C8	1.7 (4)	C3—C4—C17—C18	−71.3 (2)
C11—C12—C13—C14	−178.4 (2)		
